# Fullerene C_60_ Penetration into Leukemic Cells and Its Photoinduced Cytotoxic Effects

**DOI:** 10.1186/s11671-016-1819-5

**Published:** 2017-01-13

**Authors:** D. Franskevych, K. Palyvoda, D. Petukhov, S. Prylutska, I. Grynyuk, C. Schuetze, L. Drobot, O. Matyshevska, U. Ritter

**Affiliations:** 1 National Taras Shevchenko University of Kyiv, 64/13 Volodymyrska Street, Kyiv, 01601 Ukraine; 2 Palladin Institute of Biochemistry of the National Academy of Sciences of Ukraine, 9 Leontovicha Street, Kyiv, 01030 Ukraine; 3 Ilmenau University of Technology, 29 Ehrenbergstrasse, Ilmenau, 98693 Germany

**Keywords:** Fullerene C_60_, Photodynamic therapy, Leukemic cells, Protein tyrosine phosphorylation, Drug resistance, Mitochondrial membrane potential

## Abstract

Fullerene C_60_ as a representative of carbon nanocompounds is suggested to be promising agent for application in photodynamic therapy due to its unique physicochemical properties. The goal of this study was to estimate the accumulation of fullerene C_60_ in leukemic cells and to investigate its phototoxic effect on parental and resistant to cisplatin leukemic cells. Stable homogeneous water colloid solution of pristine C_60_ with average 50-nm diameter of nanoparticles was used in experiments. Fluorescent labeled C_60_ was synthesized by covalent conjugation of C_60_ with rhodamine B isothiocyanate. The results of confocal microscopy showed that leukemic Jurkat cells could effectively uptake fullerene C_60_ from the medium. Light-emitting diode lamp (100 mW cm^−2^, λ = 420–700 nm) was used for excitation of accumulated C_60_. A time-dependent decrease of viability was detected when leukemic Jurkat cells were exposed to combined treatment with C_60_ and visible light. The cytotoxic effect of photoexcited C_60_ was comparable with that induced by H_2_O_2_, as both agents caused 50% decrease of cell viability at 24 h at concentrations about 50 μM. Using immunoblot analysis, protein phosphotyrosine levels in cells were estimated. Combined action of C_60_ and visible light was followed by decrease of cellular proteins phosphorylation on tyrosine residues though less intensive as compared with that induced by H_2_O_2_ or protein tyrosine kinase inhibitor staurosporine. All tested agents reduced phosphorylation of 55, 70, and 90 kDa proteins while total suppression of 26 kDa protein phosphorylation was specific only for photoexcited C_60_.

The cytotoxic effect of C_60_ in combination with visible light irradiation was demonstrated also on leukemic L1210 cells both sensitive and resistant to cisplatin. It was shown that relative value of mitochondrial membrane potential measured with tetramethylrhodamine ethyl ester perchlorate (TMRE) probe was lower in resistant cells in comparison with sensitive cells and the drop of mitochondrial potential corresponded to further decrease of resistant cell viability after C_60_ photoexcitation. The data obtained allow to suggest that C_60_-mediated photodynamic treatment is a candidate for restoration of drug-resistant leukemic cell sensitivity to induction of mitochondrial way of apoptosis.

## Background

Fullerene C_60_ as a representative of a new structurally distinguished class of carbon nanocompounds is of interest for biomedical application. Its symmetrical molecule consists of 60 carbon atoms connected by sp^2^ bonds and arranged in a structure with condensed aromatic rings. The extended electron π-conjugation system determines the dual property of C_60_. High affinity of fullerene core for electron donors determines its ability to be a scavenger of free radicals. On the other hand, C_60_ molecule is able to absorb effectively UV and visible light with further transition to the first singlet excited state, then to a long-lived triplet excited state and subsequent energy transfer to molecular oxygen-yielding singlet oxygen with quantum yield close to 100% [1, 2]. Illumination of C_60_ in cell environment containing reducing agents is followed by electron transfer from fullerene triplet to molecular oxygen forming highly reactive cytotoxic superoxide and hydroxyl radicals. These unique photochemical properties of C_60_ are of particular interest for application as a photosensitizer to photodynamic therapy (PDT) of cancer. Cancer cells are metabolically active and produce high levels of reactive oxygen species (ROS); however, additional oxidative stress by exogenous ROS would result in irreversible cell damage and induction of apoptosis [3, 4].

C_60_ advantages in comparison with traditionally used photosensitizers are high absorption coefficients, high degree of photostability, little photobleaching, and prolonged response to irradiation due to the presence of multiple aromatic bonds, which are able to resist a certain interruption of π-conjugation caused by ROS back attack [5, 6].

The disadvantages of C_60_ bioapplication are its poor solubility in polar medium and heterogeneity of its aggregates in water solutions. A lot of efforts were done to improve its water solubility, including covalent modification with polar substituents and complex formation with hydrophilic polymers [7–9]. However, functionalization of fullerene molecule could be followed by decrease of photoactivation efficacy for several reasons: covalent modification of C_60_ core has been shown to change carbon atoms sp^2^ hybridization to the less-strained sp^3^ state [10]; branches of substituent could keep C_60_ structure far from the cell membrane and ROS produced could be inactivated before reaching the membrane [11]; in order to attain substantial content of C_60_ in complexes, the concentration of polymeric carrier must be high [12]; application of some polymeric micelles as carrier systems could be associated with toxic effects [13].

Nevertheless, employment of pristine C_60_ and its derivatives as photosensitizers in PDT have been shown to induce oxidative damage of membrane lipids, cleavage of DNA strands, and killing of cancer cells (HeLa and hepatoma cells) both in vitro [14] and in vivo in mouse abdominal and murine subcutaneous tumor models [5, 15]. It should be noted that in a number of works, pristine nonexcited fullerene C_60_ in concentrations up to 10^−5^–10^−4^ M was shown to be nontoxic in normal cells [12, 16, 17].

ROS attack initiated by photoexcited C_60_ is directed on multiple cellular targets both in membranes due to C_60_ high lipophilicity and in intracellular space due to C_60_ ability to penetrate plasma membrane. However, a number of issues concerning C_60_ accumulation by cancer cells of different types and biochemical mechanisms of its photocytotoxic effects remain still open.

Considering the fact that multidrug resistance of cancer cells significantly limits the efficiency of anticancer therapy, new therapeutic strategies with the use of C_60_ derivatives are developing now [18, 19]. At the same time, the cytotoxic potential of C_60_ in photodynamic therapy on drug-resistant cancer cells requires further elucidation.

The goal of this study was to estimate accumulation of fullerene C_60_ in leukemic cells and to investigate its phototoxic effect on both parental and resistant to cisplatin leukemic cells.

## Methods

### Chemicals

Staurosporine (STS), hydrogen peroxide (H_2_O_2_), MTT (3-(4,5-dimethylthiazol-2-yl)-2,5-diphenyl tetrazolium bromide), rhodamine B isothiocyanate (RITC), tetramethylrhodamine ethyl ester perchlorate (TMRE), antibodies against β-actin (1:2000 dilution), cisplatin (cis-Pt, Sigma-Aldrich Co, Ltd, USA), monoclonal antibodies against phosphotyrosine (Upstate Biotechnology Inc., USA), pyridine (Merck), and trifluoromethanesulfonic acid (Alfa Aesar) were used.

### Characterization of C_60_ Water Colloid Solution

The toluene extract obtained after graphite combustion was fractionated by gas-liquid chromatography. After toluene evaporation, fullerene C_60_ was transferred to water phase followed by prolonged ultrasound sonication (8 Hz, 8 h). Dark brown water solution of fullerene C_60_ (concentration 10^−4^ M, purity 99.5%) is highly stable (12–18 months when stored at +4 °C) molecular-colloidal system which does not contain stabilizers [20].

Using laser correlation spectroscopy, we have showed that the hydrodynamic diameter of fullerene C_60_ nanoparticles ranged from 12 to 72 nm with the mean peak position at 50 nm. No changes of fullerene C_60_ clusters diameter were detected in RPMI-1640 medium containing 5% fetal bovine serum (FBS) [21].

### Cellular Uptake of Fullerene C_60_

**Fig. 1:**
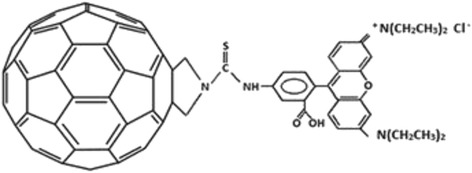
Structure of C_60_-RITC

To study the fullerene C_60_ intracellular localization in leukemic cells, the fluorescent-labeled fullerene C_60_, synthesized by covalent conjugation of C_60_ with rhodamine B isothiocyanate, was used. The precursor *N*-triphenylmethyl pyrrolidine-C_60_ was synthesized following the general strategy for the synthesis of fulleropyrrolidines [22, 23]. Exposure of trityl-fulleropyrrolidine to trifluoromethanesulfonic acid followed by addition of pyridine and rhodamine B isothiocyanate was accompanied by the attack of the carbon atom of isothiocyanate functional group by the amino group of fulleropyrrolidine giving a thiourea funtionality. Stirring in the dark at room temperature for 6 days was followed by formation of the product *N*-rhodamine-*B*-5-isothiocyanate pyrrolidine-C_60_ (C_60_-RITC) (Fig. 1).

The precipitate was washed with dichloromethane to remove any excess of RITC and dried. Ethanol water (1:2) suspension of C_60_-RITC was prepared by ultrasonication of sample for 2 min and further precipitation of insoluble moiety of the compound. The hydrolysis of the compound was slow, and suspension was stable for approximately 1 month when kept in the dark.

Leukemic cells were treated with C_60_-RITC for 2 and 18 h, washed from excess of label and fixed preparations were obtained. Intracellular content of C_60_-RITC was detected with a confocal microscope Carl Zeiss LSM 510 (Germany). Argon laser (λ = 543 nm) was used for RITC excitation. The intensity of fluorescence was estimated with the use of Zeiss LSM Image Brouser program.

### Cell Culture and Photodynamic Treatment

Leukemic cells of Jurkat and L1210 lines were obtained from the Bank of Cell Lines from Human and Animal Tissues, R. E. Kavetsky Institute of Experimental Pathology, Oncology and Radiobiology, NAS of Ukraine (Kyiv, Ukraine).

Cells were cultured in RPMI-1640 medium supplemented with 10% FBS (Sigma-Aldrich, Germany), 50 μg ml^−1^ penicillin, and 100 μg ml^−1^ streptomycin at 37 °C in a humidified atmosphere with 5% CO_2_. Cells were incubated for 2 h with or without fullerene C_60_. Photoactivation of accumulated fullerene C_60_ was done by probe irradiation in microplates with light-emitting diode lamp (LED) (420–70 nm light, irradiance 100 mW cm^−2^).

### Cell Viability (MTT) Assay

Cell viability was assessed by MTT reduction assay [24]. At indicated time points of incubation, 200 μl aliquots were removed from cell suspensions into the 96-well microplates (1 × 10^5^/well), 20 μl of MTT solution (2,5 μg ml^−1^) was added, and the plates were incubated for another 2 h. The culture medium was then replaced with 100 μl of DMSO. Diformazan formation was determined by measuring absorption at 570 nm with a plate reader (μQuant, BioTek, USA).

### Immunoblot Analysis

Immunoblotting was performed as described in [25]. Cells were washed with PBS and lysed with ice-cold lysis buffer containing complete protease inhibitor cocktail tablet. Cell lysates were clarified by centrifugation (14.000*g*, 15 min). About 30 μg of cell lysate, total protein was loaded onto a gradient 8–15% SDS-polyacrylamide gel. After electrophoresis, the proteins were transferred onto PVDF membrane and incubated with monoclonal antibody against phosphotyrosine at a dilution of 1:1000 overnight at 4 °C. The membranes were washed and incubated with goat anti-mouse peroxidase-linked secondary antibody for 1 h. Immunoreactive bands were visualized by enhanced chemiluminescence with an ECL plus Western blotting detection system (Amersham, USA). The same membranes were incubated with antibodies against β-actin (Sigma, USA, 1:2000 dilution) to provide an internal loading control.

### Mitochondrial Transmembrane Potential Aassay

Mitochondrial membrane potential was determined with fluorescent potential-sensitive probe TMRE. Cells (10^7^ ml^−1^) suspended in buffer. A consisting of (mM): KCl—5, NaCl—120, CaCl2—1, glucose—10, MgCl_2_—1, NaHCO_3_—4, HEPES—10, pH 7.4 were incubated with 100 nM TMRE for 40 min at 25 °C with addition of 0.05% Pluronic F-127, washed from excess of probe, and further incubated for different time points. TMRE fluorescence was registered with Shimadzu RF-1501 spectrofluorometer (Japan), λ_exc_ = 540 nm, λ_em_ = 595 nm. Relative values of mitochondrial potential were determined as changes in probe fluorescence after addition of protonophore FCCP (1 μM) [26].

### Statistical Analysis

The data were represented as mean ± SD of more than four independent experiments. Mean (M) and standard deviation (SD) were calculated for each group. Statistical analysis was performed using two-way ANOVA followed by post Bonferroni tests. A value of *p* < 0.05 was considered statistically significant. Data processing and plotting were performed by IBM PC using specialized applications GraphPad Prism 7 (GraphPad Software Inc., USA) and Gel-Pro Analyzer 6.3 (Media Cybernetics Inc., USA).

## Results and Discussion

**Fig. 2:**
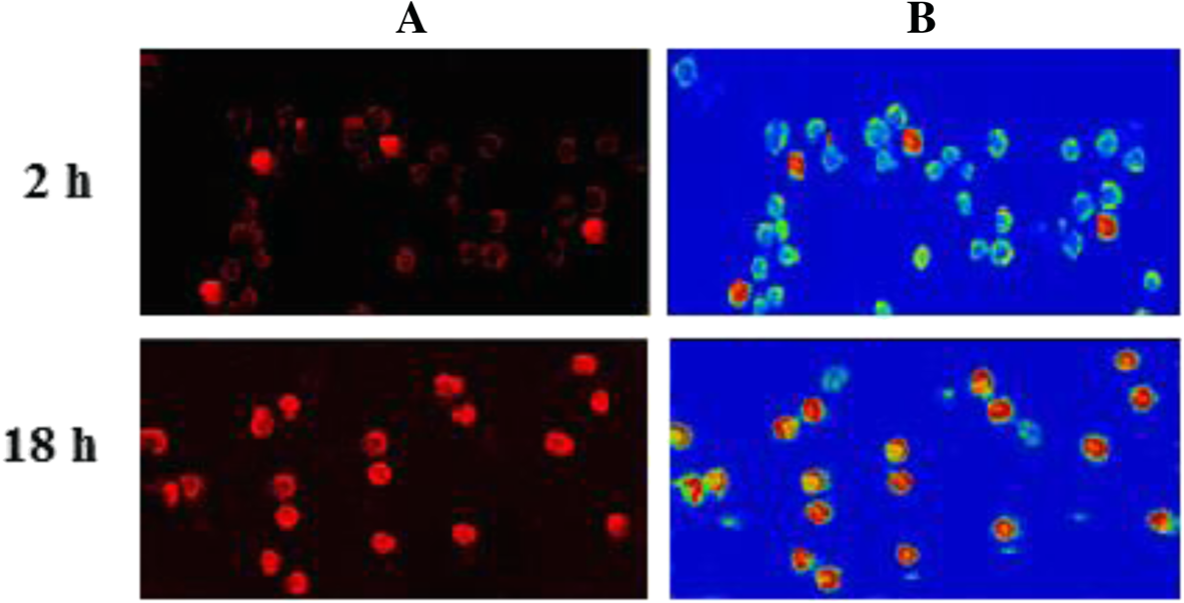
Confocal microscopy images (**a**) and fluorescence intensity (**b**) of Jurkat leukemic cells incubated with fullerene C_60_-RITC

High lipophilicity of fullerene C_60_ molecule determines its affinity to membrane lipid bilayer and ability to penetrate cell plasma membrane. It is suggested that interaction of C_60_ cluster with membrane is followed by disaggregation of nC_60_ within bilayer and diffusion of molecules through transient micropores [27]. To estimate the uptake of C_60_ by leukemic cells, we used the fluorescent-labeled C_60_ obtained by covalent conjugation of C_60_ with rhodamine B isothiocyanate (C_60_-RITC). The conjugate was proved to be a nice fluorescent probe for detection and monitoring of C_60_ nanoparticles accumulation in the cell. Shown in Fig. 2 are the confocal fluorescence images of leukemic cells in dynamics of incubation with C_60_-RITC. It can be seen that after 2 h of incubation, the plasma membrane and the cytoplasm in most of the cells in population appear stained. Intracellular fluorescence intensity of C_60_-RITC was further increased at 18 h indicating than no leakage of accumulated nanostructure occurs. The data obtained confirm that leukemic cells could effectively uptake fullerene C_60_ from the medium.

Our data are in agreement with studies where the uptake of pristine C_60_ in nano-aggregated form by MCF10A normal breast epithelial cells [16], lung epithelial adenocarcinoma A549 cells [28], and leukemic monocyte macrophage RAW 264.7 cells [29] was demonstrated.

Taking into account that the dynamics of C_60_ uptake by leukemic cells is delayed while simple diffuse through the plasma membrane is a rapid process; it could be postulated that C_60_ nanoparticles are taken up by slower process of adsorptive endocytosis [5, 30]. In further experiments, a 2-h incubation of cells with fullerene C_60_ was used.

**Fig. 3:**
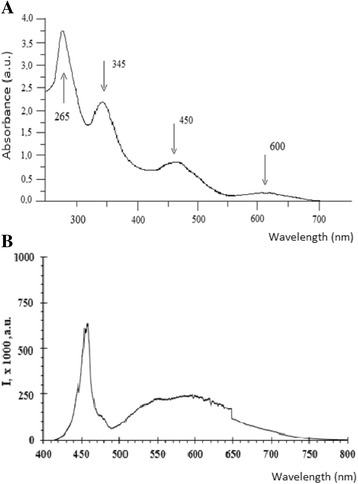
Absorption spectra of fullerene C_60_ in water suspension (10^−4^ M) (**a**) and emission spectrum of light-emitting diode lamp (**b**) used for C_60_ photodynamic treatment of leukemic cells

Efficiency of fullerene C_60_ photoactivation largely depends on the characteristics of the molecule optical absorption. In Fig. 3a, the spectrum of optical absorption of fullerene C_60_ in water colloid suspension is shown. The highest absorption is observed in the ultraviolet (265 nm, 345 nm) range; two peaks which are less in magnitude are located in blue (450 nm) and red (600 nm) regions of the visible light. UV waves are unfavorable in cancer therapy because they do not possess the ability to penetrate deeply into tissue and besides induce damaging effects. Therefore, LED lamp with emission range in the visible region of the spectrum of 410 –700 nm was used for C_60_ photoexcitation. Emission spectrum of this lamp showed the peak at 450 nm and extremum in the range of 550–600 nm (Fig. 3b), which coincide with maxima in absorption spectra of fullerene C_60_ in visible region.

Despite the common notion that photodamaging effect of C_60_ on cancer cells is determined by effective production of cytotoxic ROS in intracellular space [31, 32], the biochemical pathways and signaling networks of its realization still need to be elucidated.

Increased level of protein phosphorylation on tyrosine residues caused by enhanced activity of phosphotyrosine kinases (PTK) due to mutations, overexpression, and autocrine-paracrine stimulation is one of the hallmarks of uncontrolled cells proliferation and malignancy. Application of drugs able to induce apoptosis by PTK inactivation seems to be promising for antileukemic therapy [33]. Protein tyrosine phosphorylation events involved in signal transfer is shown to be regulated by ROS, which could directly oxidize specific cysteine residues in protein tyrosine phosphatases or cross-talk indirectly with upstream regulatory mechanisms. Taking into account these facts, the comparative experimental study of viability and protein phosphotyrosine (pTyr) status of Jurkat cells treated with photoexcited fullerene C_60_, staurosporine (a broad-acting protein kinases inhibitor), or hydrogen peroxide (inducer of oxidative stress) was done.

**Fig. 4:**
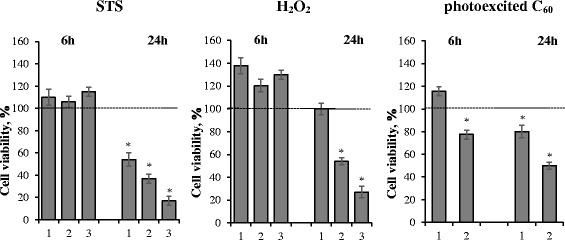
Jurkat cell viability after treatment with STS (*1*—0.01 μM, *2*—0.1 μM, 3—1 μM STS); H_2_O_2_ (*1*—25 μM, *2*—50 μM, *3*—100 μM H_2_O_2_); photoexcited fullerene C_60_ (*1*—5 μM, *2*—50 μM C_60_). *p < 0.05 in comparison to control

The data on viability of leukemic cells under used experimental conditions are presented in Fig. 4. It should be noted that no statistically valid changes in viability of leukemic cells subjected to irradiation with visible light or fullerene C_60_ separately were detected (data not shown). Only in the case of their combined action dose—dependent decrease of cell viability was detected at 24 h. Approximately, 50% decrease of cell viability at 24 h was induced by comparatively equal concentrations (about 50 μM) of both photoexcited C_60_ and H_2_O_2_, while for STS, the index was much lower (0.01 μM) (Fig. 4). In this connection, it must be noted that cytotoxic effect of STS on normal cardiomyocytes [34] and hepatocytes [35] appeared to be a limiting factor in STS application for anti-tumor therapy.

**Fig. 5:**
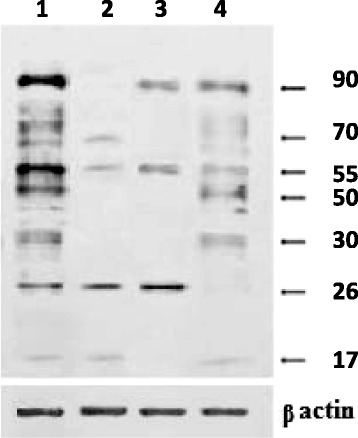
Western blot analysis of protein phosphotyrosine level in Jurkat cells at 24 h of incubation in control (*1*) and after treatment with 0.01 μM STS (*2*), 50 μM H_2_O_2_ (*3*), photoexcited fullerene C_60_ (*4*)

The next task was to estimate leukemic cell protein phosphotyrosine status as the systemic marker of PTK-dependent uncontrolled proliferation. The main immunoreactive bands detected by monoclonal antibodies against phosphotyrosine correspond to proteins with molecular weight in the range of 17, 26, 30, 50, 55, 70, and 90 kDa (Fig. 5, track 1) confirming intense protein tyrosine phosphorylation in leukemic cells. All investigated agents induce decrease in protein phosphotyrosine level in leukemic cells, but differences in intensity of decrease and in pattern of proteins with decreased pTyr level were observed.

Treatment with STS was followed by total suppression of 30, 50, and 90 kDa protein phosphorylation, trace-level phosphorylation of bands corresponding to 17, 55, and 70 kDa proteins was detected (Fig. 5, track 2). Treatment with H_2_O_2_ was followed by total dephosphorylation of 17, 30, 50, and 70 kDa proteins, as well as reduced level of 55 and 90 kDa protein modification (Fig. 5, track 3). Modification of 26 kDa protein in cells exposed to STS appeared to remain at control level and in cells treated with H_2_O_2_ to be even higher than in control.

Combined action of fullerene C_60_ (50 μM) and visible light was followed in general by less intensive decrease of protein phosphotyrosine level in cells as compared with that induced by STS or H_2_O_2_. The similar effects of all tested agents (reduced phosphorylation of 55, 70, and 90 kDa proteins) were observed, while total suppression of 26 kDa protein phosphorylation was specific only for photoexcited C_60_. Based on the data obtained, we suggest that 26 kDa protein could potentially participate in mechanisms that mediate effect of photoexcited C_60_ on leukemic cell survival.

The data obtained show that inhibition of protein tyrosine phosphorylation could be involved into realization of C_60_ photocytotoxic effects along with modification of other signaling mechanisms and involvement of different targets.

**Fig. 6:**
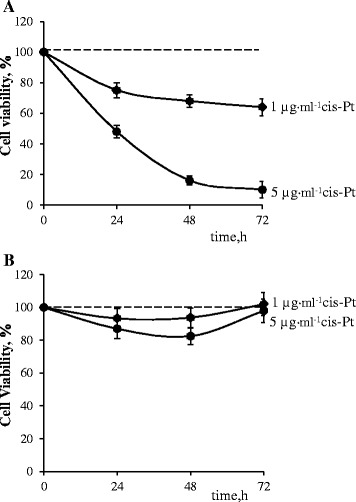
Viability of L1210 (**a**) and L1210R (**b**) cells incubated in the presence of cisplatin

Considering the fact that the efficiency of anticancer therapy is substantially limited by development of cancer cells multidrug resistance, the photodynamic potential of fullerene C_60_ was estimated in further experiments on two leukemic cell lines—parental (L1210) and resistant to cisplatin (L1210R). The data on cell survival after treatment with 1 and 5 μg ml^−1^ of cisplatin as indicator of drug sensitivity are presented in Fig. 6.

The viability of parental L1210 cells treated with cisplatin was decreased in a dose and time-dependent manner. At 24 h of incubation, the viability of L1210 cells treated with 1 μg ml^−1^ was decreased by 30% and with 5 μg ml^−1^—by 50%, no cell survival was detected at 72-h incubation with 5 μg ml^−1^ of cisplatin (Fig. 6a).

Treatment of L1210 resistant cells with cisplatin in both concentrations has no effect on cell viability. Minor decrease in viability of resistant cells treated with 5 μg ml^−1^ at 48 h was restored at 72 h (Fig. 6b).

**Fig. 7:**
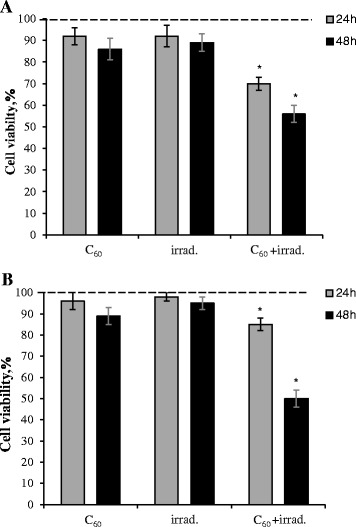
Viability of L1210 (**a**) and L1210R (**b**) cells exposed to fullerene C_60_, visible light irradiation, or their combined action. *p < 0.05 in comparison to control

No statistical decrease of L1210 or L1210R cell viability at 24 and 48 h after treatment with C_60_ (50 μM) or light irradiation per se was observed (Fig. 7). But after combined treatment with fullerene C_60_ and light time-dependent decrease of parental, L1210 cell viability (to 70 and 55% at 24 and 48 h, respectively) was observed (Fig. 7a). It should be noted that phototoxic effect of C_60_ was detected not only in parental but in cisplatin resistant cells as well. At 48 h of incubation, the decrease of L1210R cell viability reached 50% (Fig. 7b) that was similar to C_60_-mediated photodamaging effect on parental leukemic cells.

Development of multidrug resistance (MDR) in cancer cells is known to be accompanied by the overexpression of ATP-binding cassette (ABC) transporters, which provide efficient extrusion of drugs across plasma membrane into extracellular space thereby decreasing intracellular drug concentration [36]. The inhibitory action of nanoparticles on ABC transporter activity due to their direct or indirect interactions was suggested. Efficient accumulation of pristine fullerene C_60_ in both MDR and drug-sensitive human leukemia cells and incapability of P-gp type of ABC transporters to extrude C_60_ nanoparticles was demonstrated in [37] as well as our data confirm this suggestion. So, we assume that phototoxic effect of fullerene C_60_ on cisplatin resistant L1210 cells could be mediated by its efficient intracellular accumulation.

Evaluation of the early effects involved in activation of death signaling pathways is a necessary step in determining the mechanisms of photoexcited fullerene C_60_ long-term cytotoxicity. Taking into account that mitochondrial transmembrane potential (∆ψ) dissipation is an important early marker of apoptotic cell death induction, we estimated the relative value of mitochondrial membrane potential in L1210 and L1210R cells at 3 h after photoexcitation of accumulated fullerene C_60_.

**Fig. 8:**
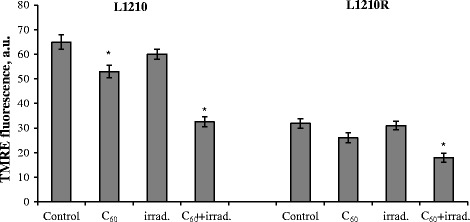
Relative value of mitochondrial membrane potential in L1210 and L1210R cells exposed to fullerene C_60_, visible light irradiation, or their combined action. *p < 0.05 in comparison to control

The difference between relative values of mitochondrial potential in parental and cisplatin-resistant leukemic cells was demonstrated (Fig. 8). Intensity of TMRE signal in L1210R was lower than that in L1210 cells. These results are in agreement with data concerning decreased activity of electron-transport chain and lower hyperpolarization of mitochondrial inner membrane in drug-resistant cancer cells compared to parental [38]. Leukemic cells resistant to cisplatin are shown to express increased level of uncoupling protein-2 (UCP2) and to maintain reduced ΔΨ in comparison with cisplatin-sensitive cells, ensuring by this way reduced ROS production and resistance to cisplatin-induced apoptosis [39].

It was shown that irradiation in visible spectrum did not change mitochondrial transmembrane potential in cells of both lines (Fig. 8). After treatment with fullerene C_60_, the relative value of mitochondrial membrane potential in L1210 cells was decreased presumably due to C_60_ interaction with mitochondrial membranes and permeation into intermembrane space. It has been proposed that the negative surface charge of hydrated fullerene C_60_ clusters promotes binding to mitochondrial membranes with further dissipation of proton gradient [40].

Combined treatment with fullerene C_60_ and irradiation was followed by strongly pronounced decrease of TMRE fluorescent signal not only in L1210 but also in L1210R cells. The relative value of mitochondrial membrane potential in L1210 and L1210R cells decreased by 2 and 1.6 times as compared with control values, respectively.

Considering the fact that our previous study has demonstrated substantial increase of ROS production in L1210 cells after pristine fullerene C_60_ photoexcitation in UV/Vis spectrum [41], we suggested that C_60_ interaction with mitochondrial membranes and ROS generation after its photoexcitation are followed by essential uncoupling of electron-transporting chain components, proton leak across inner mitochondrial membrane, and Δψ drop.

Summarizing, we assume that photoexcitation of accumulated fullerene C_60_ with visible light could induce cell death of parental and resistant to cisplatin leukemic cells by ROS-dependent mitochondrial way.

## Conclusions

In this study, stable homogenous water colloid solution of pristine fullerene C_60_ was used in experiments for estimation of C_60_ photocytotoxicity on leukemic cells. C_60_-RITC conjugate was synthesized for monitoring C_60_ entry into leukemic cells by confocal microscopy. Leukemic Jurkat cells could uptake fullerene C_60_ from the medium and retain the accumulated nanoparticles over 18 h. Combined treatment with C_60_ and visible light is followed by time-dependent decrease of Jurkat cell viability. Inhibition of protein tyrosine phosphorylation could be one of the mechanisms of C_60_ photocytotoxic effects realization.

C_60_-mediated photodynamic effect was demonstrated also on leukemic L1210 cells both sensitive and resistant to cisplatin. Decrease of drug-resistant cell viability after C_60_ photoexcitation corresponded to a significant drop of mitochondrial potential. The data obtained allow to suggest that C_60_ photodynamic treatment might be a potential strategy for restoring the drug-resistant leukemic cell sensitivity to induction of mitochondrial way of apoptosis.

## Abbreviations

∆ψ: Mitochondrial transmembrane potential
ABC transporters: ATP-binding cassette transporters
cis-Pt: Cisplatin
DMSO: Dimethyl sulfoxide
FBS: Fetal bovine serum
FCCP: Carbonyl cyanide-*4*-(trifluoromethoxy)phenylhydrazone
LED: Light-emitting diode lamp
MDR: Multidrug resistance
MTT: 3-(4,5-Dimethyl-2-thiazolyl)-2,5- diphenyl-2-H-tetrazolium bromide
PBS: Phosphate buffer solution
PDT: Photodynamic therapy
PDT: Photodynamic therapy
PTK: Phosphotyrosine kinase
pTyr: Phosphotyrosine
RITC: Rhodamine B isothiocyanate
ROS: Reactive oxygen species
RPMI-1640 medium: Roswell Park Memorial Institute medium
STS: Staurosporine
TMRE: Tetramethylrhodamine ethyl ester perchlorate
